# CCL2 Inhibitor Bindarit Improve Postoperative Cognitive Function by Attenuating Pericyte Loss-Related Blood–Brain Barrier Disruption and Neuroinflammation

**DOI:** 10.1155/mi/7248780

**Published:** 2025-06-12

**Authors:** Hui Yuan, Bo Lu, Daofan Sun, Junping Chen, Xiangming Fang

**Affiliations:** ^1^Department of Anesthesiology, The First Affiliated Hospital, Zhejiang University School of Medicine, Hangzhou 310052, Zhejiang Province, China; ^2^Department of Anesthesiology, Ningbo NO. 2 Hospital, Ningbo 315010, Zhejiang Province, China

**Keywords:** blood–brain barrier, CCL2, neuroinflammation, pericyte, perioperative neurocognitive disorder

## Abstract

Perioperative neurocognitive disorder (PND) is a common complication in elderly patients undergoing surgery and anesthesia and associated with the impaired recovery. Previous studies have demonstrated that PND was correlated with the pericyte (PC) loss in brain, but the underlying mechanisms remain unclear. This study investigates whether C–C motif chemokine ligand 2 (CCL2) in hippocampal tissues contributes to postoperative PC injury, blood–brain barrier (BBB) disruption, neuroinflammation, and cognitive dysfunction. Sixteen-month-old C57BL/6 mice underwent tibial fracture surgery to induce PND. CCL2 expression in hippocampal tissues was downregulated using intraperitoneal injections of 200 mg/kg daily Bindarit for 4 days prior to surgery. Behavioral tests were conducted on the third day postsurgery and brain tissues were collected. Western blotting assessed CCL2 expression in the hippocampus, immunofluorescence evaluated PC coverage, BBB integrity, and neuroinflammation, and transmission electron microscopy (TEM) examined BBB microstructure. Bindarit effectively inhibited the surgery-induced increase in hippocampal CCL2 expression and improved postoperative cognitive function. Behavioral tests, including the open field (OF) test and novel object recognition (NOR) test, indicated enhanced locomotor activity and short-term memory in Bindarit-treated mice compared to controls. Immunofluorescence analysis revealed that Bindarit treatment mitigated the reduction in capillary length and tight junction (TJ) protein expression, specifically claudin-5 and occludin, which was seen with decreased PC coverage. Additionally, Bindarit suppressed the activation of hippocampal microglia and astrocytes, as evidenced by reduced Iba-1 and GFAP staining. TEM analysis confirmed that Bindarit preserved BBB microstructure integrity postsurgery. This study demonstrates that the CCL2 inhibitor Bindarit significantly reduces the incidence of PND by preventing PC loss, thereby protecting the BBB and alleviating neuroinflammation. These findings suggest that targeting CCL2 could be a potential therapeutic strategy for preventing and treating PND.

## 1. Introduction

Perioperative neurocognitive disorder (PND) is a frequent and serious complication in elderly patients undergoing surgery and anesthesia, often leading to impaired postoperative recovery and significantly diminishing quality of life [1, 2]. Despite extensive research, the underlying mechanisms of PND remain poorly understood. Surgery have been shown to exacerbate the disruption of the blood–brain barrier (BBB) in the aging brain, thereby promoting neuroinflammation—a pivotal factor in the development of PND[3, 4]. Thus, understanding how surgery affects BBB integrity is essential.

Pericytes (PCs), integral to maintaining BBB structure, play a critical role in regulating its permeability by covering vascular walls [5]. Impairments in PC function or quantity can lead to a decrease in the secretion of tight junction (TJ) proteins, increased BBB permeability, and destabilization of the central nervous system (CNS) microenvironment [6]. Recent studies have demonstrated that orthopedic surgery reduces the expression of the PC marker CD13 in the hippocampus [7]. Our findings further indicate that postoperative BBB breakdown, neuroinflammation, and cognitive deficits in elderly mice are associated with reduced PC coverage [8]. Exploring the underlying causes of reduced PC coverage postsurgery could provide insights into the prevention and management of PND.

C–C motif chemokine ligand 2 (CCL2), also known as monocyte chemoattractant protein-1 (MCP-1), plays a critical role in BBB disruption and neuroinflammation across various brain disorders [9]. Studies have demonstrated increased CCL2 expression in the hippocampus following surgery, correlating with microglial activation, neuronal damage, and cognitive decline [10]. Whereases brain PCs are the major to secrete it in response to systemic inflammatory stimuli and further lead to the cytokine storm [11]. Therefore, PC-derived CCL2 might drive postoperative BBB destruction and neuroinflammation. Evidence linking CCL2 levels to retinal PC injury suggests a possible connection between postoperative BBB damage, neuroinflammation, and PC injury due to CCL2 overexpression following surgery [12]. Although Xu et al. [10] highlighted the involvement of hippocampal CCL2/CCR2 in postoperative cognitive dysfunction using CCR2 antagonists, the specific impact of CCL2 on PND, particularly concerning its effects on the BBB and PC, remains inadequately addressed.

In this study, we employed a CCL2 inhibitor to delineate the role of CCL2 in postoperative PC injury and BBB disruption, aiming to explore the mechanisms underlying postoperative neuroinflammation and cognitive dysfunction.

## 2. Materials and Methods

### 2.1. Animals

Sixteen-month-old male C57BL/6 mice served as experimental subjects. All animals were housed in a room with controlled temperature and humidity, undergoing a 12-h day–night cycle. All procedures received approval from the Animal Care and Use Committee of Ningbo University (2020-250), adhering to the National Institutes of Health guidelines for the Care and Use of Laboratory Animals.

### 2.2. Anesthesia and Surgery

The anesthesia and surgical procedures were consistent with our established protocols [8, 13]. Briefly, after inducing anesthesia with isoflurane, the lower left limb was shaved and disinfected. An incision was made through the skin and muscles above the tibia, exposing its upper surface. A small hole was drilled using a burr and a 0.38 mm steel needle was inserted. The tibia was then fractured at one-third of its length to mimic tibial fracture fixation surgery. After the procedure, the incision was sutured and an ointment containing erythromycin and ropivacaine was applied for anti-infection and analgesia.

### 2.3. Drug Administration and Experimental Procedure

**Figure 1:**
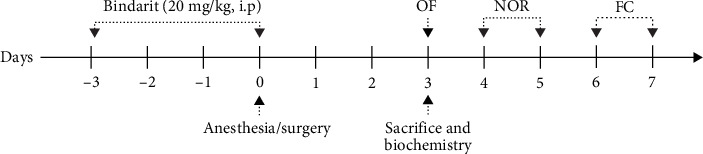
Schematic diagram of the experimental procedures. The Bindarit group received intraperitoneal injections of Bindarit (20 mg/kg) daily for 4 days prior to surgery. Postsurgery, one set of mice was sacrificed on Day 3 for biochemical analysis. A separate set of mice underwent behavioral tests: the open field (OF) test on Day 3 to assess locomotor activity and anxiety, the novel object recognition (NOR) test on Days 4 and 5 to evaluate short-term memory, and the fear conditioning (FC) test on Days 6 and 7 to assess contextual and cued fear memory.

To investigate the role of CCL2 in postoperative PC injury, BBB disruption, neuroinflammation, and cognitive dysfunction, Bindarit was administered to inhibit CCL2 overexpression postsurgery. Following established methodology [14], Bindarit was dissolved in 0.5% methylcellulose at 20 mg/ml. The Bindarit group received intraperitoneal injections of Bindarit (200 mg/kg) daily for 4 days prior to surgery, while control mice received equivalent doses of methylcellulose. The final injection was administered 30 min before the tibial fracture surgery. The experimental procedure is depicted in Figure 1.

### 2.4. Behavioral Tests

Behavioral assessments were conducted on postoperative Day 3. Tests were performed within the same time frame and according to methods described in previous studies [8]. The experimental process is illustrated in Figure 1.

#### 2.4.1. Open Field (OF) Test

The OF test was used to measure the locomotor activity and anxiety levels in mice, as detailed in our prior research [8, 15]. On postoperative Day 3, mice were introduced into a 40 × 40 × 40 cm^3^ OF box, divided into edge and central area (20 × 20 cm^2^). Each mouse was consistently placed in the same starting position and allowed to freely explore for 5 minutes. Mice activity trajectories were recorded and analyzed using VisuTrack software (Xinruan, Shanghai, China). Locomotor activity was gauged by average speed and anxiety levels were assessed by the time and distance spent in the central area.

#### 2.4.2. Novel Object Recognition (NOR) Test

The NOR test, which evaluates visual and spatial short-term memory, involves rodents displaying a preference for novel objects over familiar ones. This methodology is consistent with our previous descriptions [8, 15, 16]. On postoperative Day 4, during the familiarization phase, mice were allowed to explore two identical square objects (A) in a 40 × 40 cm^2^ area for 5 min. Twenty-four hours later, in the testing phase, one of these objects was replaced with a new circular object (B) and exploration was permitted for another 5 min. The time spent exploring objects A and B was recorded with VisuTrack software. The recognition index (RI) was calculated as follows: RI = time spent exploring the novel object (B)/total exploration time of both objects (A + B).

#### 2.4.3. Fear Conditioning (FC) Test

FC, a method for assessing learning and memory, followed the protocol outlined in our published studies [8, 13, 16]. On postoperative Day 6, mice were placed in a dark chamber (30 × 30 × 45 cm^3^) with a conditioned stimulus apparatus (Supercs, Xinruan) and allowed to explore for 180 s. A 30-s auditory stimulus (70 dB, 3 kHz) followed. Immediately after the tone, a 2-s foot shock (0.75 mA) was administered and the process was repeated after 60 s. After 24 h, the mice were returned to an unstimulated dark chamber for 3 min to measure contextual fear memory by recording the freezing time with supercs software. Two hours later, they were placed in a novel environment and exposed to the same auditory cue to assess cued fear memory.

### 2.5. Immunofluorescence Staining

Following established protocols, mice were euthanised by intraperitoneal injection of 3% pentobarbital at a dose of 100 mg/kg, followed by cervical dislocation on the third day postsurgery. Brain tissues were collected and immediately fixed in 4% PFA, followed by sequential immersion in 15% and 30% sucrose solutions. The brains were then sectioned into 25 μm thick slices. These sections were incubated in 0.1 M phosphate-buffered saline (PBS) containing 0.5% Triton X-100 along with the following primary antibodies: fluorescein-conjugated tomato lectin (1:200, FL-1171, Vector), anti-CD13 (1:100, AF2335, RD), anti-claudin 5 (1:100, 35–2500, Thermo Fisher), anti-occludin (1:250, 33–1500, Thermo Fisher), anti-Iba-1 (1:100, ab178846, Abcam), and mouse anti-GFAP (1:300, 3670, CST), and were left to incubate overnight at 4°C. Afterward, the sections were rinsed three times with PBS and exposed to secondary antibodies: Cy3-conjugated goat anti-mouse (1:500, ab97035, Abcam), Alexa Fluor 488-conjugated goat anti-rabbit (1:500, ab150077, Abcam), and Alexa Fluor 488-conjugated goat anti-mouse (1:500, ab150113, Abcam). The slices were then imaged using a confocal laser scanning microscope (SP8, Leica, Frankfurt, Germany).

### 2.6. Confocal Microscopy Analysis of BBB Related Indicators

In line with our previous research [8], all acquired images were 320 μm × 320 μm in size, with a maximum *z*-projection of 5 μm. Four hippocampal images were randomly taken from two nonadjacent slices per sample and then averaged. All images were analyzed with Image-Pro Plus 7 and technicians blinded to the experimental groups conducted the image acquisition and analysis.

#### 2.6.1. CD13-Positive PC Coverage and Capillary Length

Double immunostaining with CD13 and endothelial-specific lectin was used to analyze PC coverage of brain capillaries, following our earlier methodologies [5, 6, 8, 17–19]. Analysis was performed using Image-Pro Plus 7 software. PC coverage was calculated as the percentage of CD13-positive area relative to the lectin-positive area, while capillary length was measured as the length (mm) of lectin-positive endothelial cells [8, 18].

#### 2.6.2. Length of Claudin-5 and Occludin

The lengths of occludin and claudin-5 immunofluorescence within the lectin-positive capillary area were analyzed using Image-Pro Plus 7 software. The lengths of occludin and claudin-5 were quantified in millimeters per mm^2^ of lectin-positive capillary surface area [8].

### 2.7. Western Blot Analysis

The procedure mirrored the methodologies outlined in our previous studies [13]. Hippocampal tissues were homogenized in RIPA buffer, lysed at 4°C, and centrifuged at 12,000 × *g* for 25 min. Proteins from the supernatant were loaded equally, separated by gel electrophoresis, and then, transferred to PVDF membranes. The membranes were blocked for 60 min and subsequently incubated overnight at 4°C with primary antibodies: rabbit anti-CCL2 (1:1000, ab214819, Abcam) and rabbit anti-β-actin (1:3000, GB15003, Servicebio). Following three PBST washes, the membranes were incubated with goat anti-rabbit IgG (1:5000, GB23303, Servicebio). Immunoreactive bands were detected using an enhanced chemiluminescence kit and quantified by densitometry.

### 2.8. Transmission Electron Microscopy (TEM)

TEM was utilized to observe structural changes in the BBB. On postoperative Day 3, hippocampal tissues were harvested, fixed in glutaraldehyde, sectioned into 1 mm^3^ pieces, and further fixed with 1% osmium tetroxide for 3 h. The tissues were then washed three times with PBS, dehydrated in ascending concentrations of ethanol, and embedded in epoxy resin. Ultrathin sections (60–80 nm) were stained with uranyl acetate and lead citrate for 15 min each and air-dried overnight at room temperature. The sections were examined under TEM and images were captured for analysis.

### 2.9. Activation Levels of Microglia and Astrocytes

Confocal microscopy was performed with a uniform image size of 600 μm × 600 μm. Following established protocols [13, 16, 17], the activation levels of microglia and astrocytes were indicated by the percentage of area occupied by Iba-1 or GFAP-positive cells. Images were analyzed using ImageJ software, adjusting for background and setting thresholds to calculate the Iba-1 or GFAP-positive areas. Experimenters handling image acquisition and quantification were blinded to the experimental groups.

### 2.10. Statistical Analysis

Statistical analysis was performed using SPSS 22.0. Data are expressed as mean ± standard error of the mean (SEM). One-way analysis of variance (ANOVA) was applied for data analysis, followed by Bonferroni's post hoc test and Pearson correlation analysis. A *p*-value of less than 0.05 was considered statistically significant.

## 3. Results

### 3.1. Bindarit Inhibited the Overexpression of CCL2 in the Hippocampus of Aged Mice After Anesthesia/Surgery

**Figure 2:**
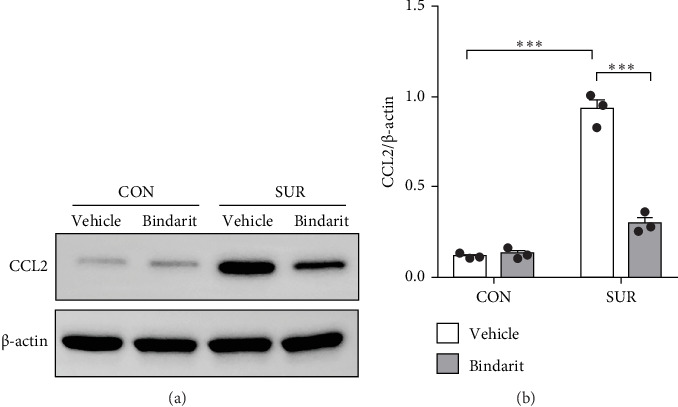
Bindarit reduced CCL2 expression in the hippocampus after anesthesia/surgery. (A) Immunoblotting of CCL2 expression in hippocampus. (B) Quantitative analysis of gray value of CCL2 immunoblotting. Data are presented as mean ± SEM (*n* = 3), *⁣*^*∗∗∗*^*p*  < 0.001.

To assess the effect of Bindarit on CCL2 expression, we utilized western blot analysis to measure hippocampal CCL2 levels. CCL2 expression in the surgery plus vehicle (SUR + Vehicle) group was significantly elevated compared to the control plus vehicle (CON + Vehicle) group (Figure 2). Conversely, CCL2 levels in the surgery plus Bindarit (SUR + Bindarit) group were significantly reduced compared to the SUR + Vehicle group (Figure 2), demonstrating that Bindarit effectively inhibits the overexpression of CCL2.

### 3.2. Bindarit Alleviated Cognitive Impairment in Aged Mice After Anesthesia/Surgery

**Figure 3:**
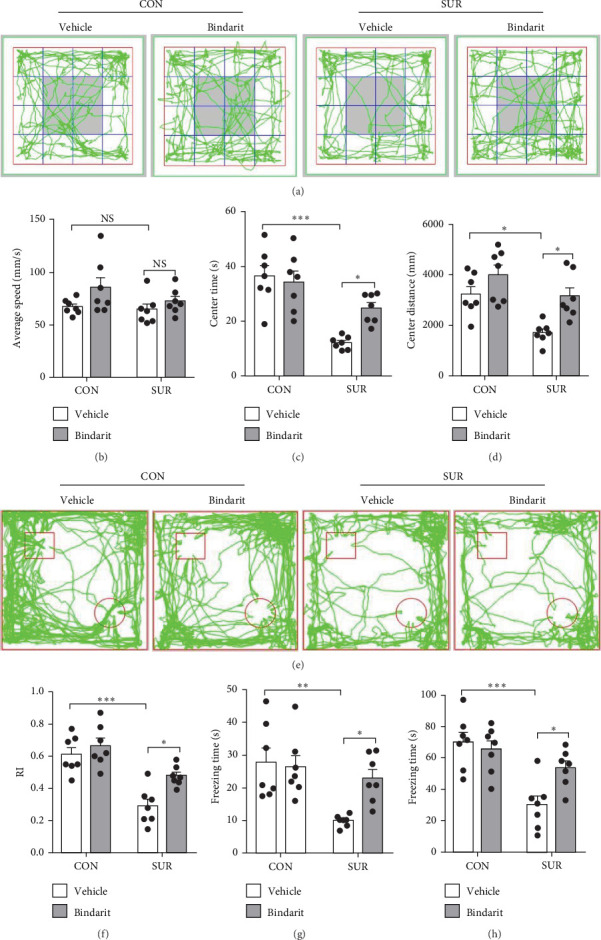
Bindarit alleviated anesthesia/surgery induced cognitive impairment. (A) Representative images of motion trajectories in the OF test. (B) Average speed of mice in the OF test. (C) Activity time of mice in the central area during the OF test. (D) Distance moved by mice in the central area during the OF test. (E) Representative images of motion trajectories in the NOR test. (F) RI of mice in the NOR test. (G) Freezing time during contextual fear memory test. (H) Freezing time during cued fear memory test. Data are presented as mean ± SEM (*n* = 7), *⁣*^*∗*^*p*  < 0.05, *⁣*^*∗∗*^*p*  < 0.01, *⁣*^*∗∗∗*^*p*  < 0.001.

In the OF test, no differences were observed in average speed among the four groups of mice (Figure 3A,B), suggesting that motor functions were restored by the third day postsurgery. The SUR + Vehicle group exhibited a significant reduction in both time and distance spent in the central area compared to the CON + Vehicle group (Figure 3C,D), indicating increased anxiety. However, the SUR + Bindarit group showed a significant increase in time spent in the central area compared to the SUR + Vehicle group (Figure 3C,D), suggesting reduced anxiety levels.

In the NOR test, the RI for the SUR + Vehicle group was significantly lower than that of the CON group, while the SUR + Bindarit group showed a significantly higher RI compared to the SUR + Vehicle group (Figure 3E,F). Furthermore, in the FC test, the SUR + Vehicle group displayed markedly reduced freezing times for both contextual and cued fear memory *compared* to the CON + Vehicle group (Figure 3G,H). In contrast, the SUR + Bindarit group exhibited significantly increased freezing times compared to the SUR + Vehicle group (Figure 3G,H). These findings indicate that Bindarit significantly mitigates PND in aged mice following anesthesia and surgery.

### 3.3. Bindarit Reversed PC Loss and Microvascular Degeneration in the Hippocampus of Aged Mice Following Anesthesia/Surgery

**Figure 4:**
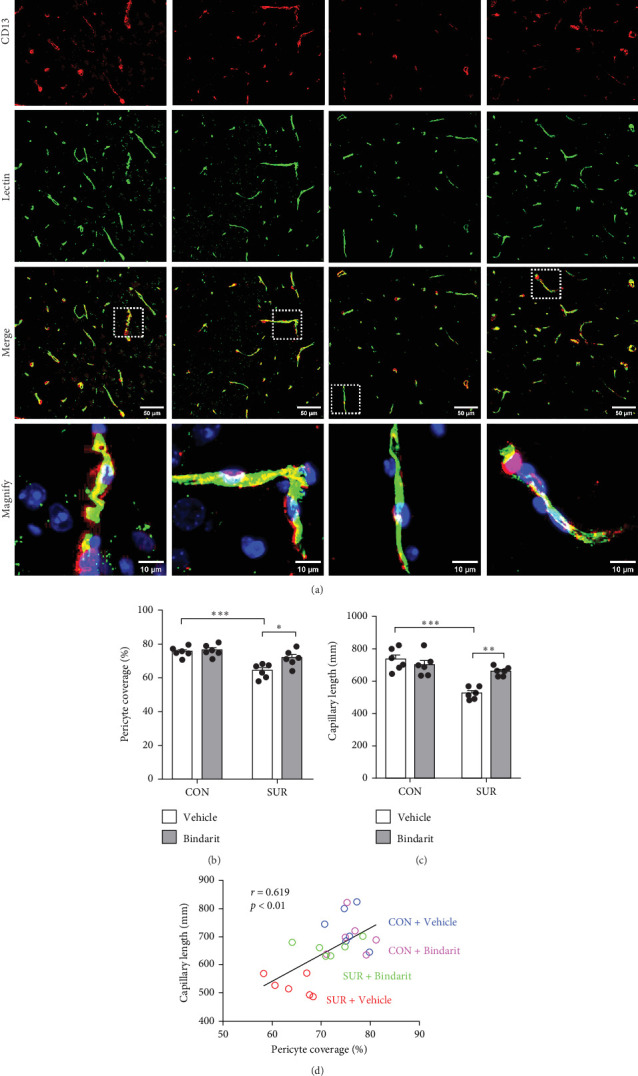
Bindarit inhibited the loss of PC and reduction of capillary length induced by anesthesia/surgery. (A) Representative confocal images of CD13-positive PC (red) and lectin-positive capillary (green) in the mice hippocampus. (B) Quantitative analysis of CD13-positive PC coverage. (C) Quantitative analysis of lectin-positive capillary length. (D) Correlation analysis between PC coverage and capillary length. Data are presented as mean ± SEM (*n* = 6), *⁣*^*∗*^*p*  < 0.05, *⁣*^*∗∗*^*p*  < 0.01, *⁣*^*∗∗∗*^*p*  < 0.001.

PCs are pivotal in mediating the functionality of brain capillaries and are the primary and most prolific producers of CCL2 during neuroinflammatory responses in the brain [6, 8, 11]. To assess Bindarit's impact on PCs and capillaries, we conducted double immunofluorescence staining to evaluate the distribution of CD13-positive PCs and lectin-positive brain capillaries in the hippocampus across different experimental groups (Figure 4A). The results revealed a significant reduction in PC coverage and capillary length in the SUR + Vehicle group compared to the CON + Vehicle group (Figure 4B,C). In contrast, mice in the SUR + Bindarit group showed significant increases in both PC coverage and capillary length relative to the SUR + Vehicle group (Figure 4B,C). Pearson correlation analysis indicated a significant association between the reduction in hippocampal capillary length and PC loss in aged mice following anesthesia/surgery (Figure 4D).

### 3.4. Bindarit Relieved the Loss of BBB-Related TJ Proteins in the Hippocampus of Aged Mice After Anesthesia/Surgery

**Figure 5:**
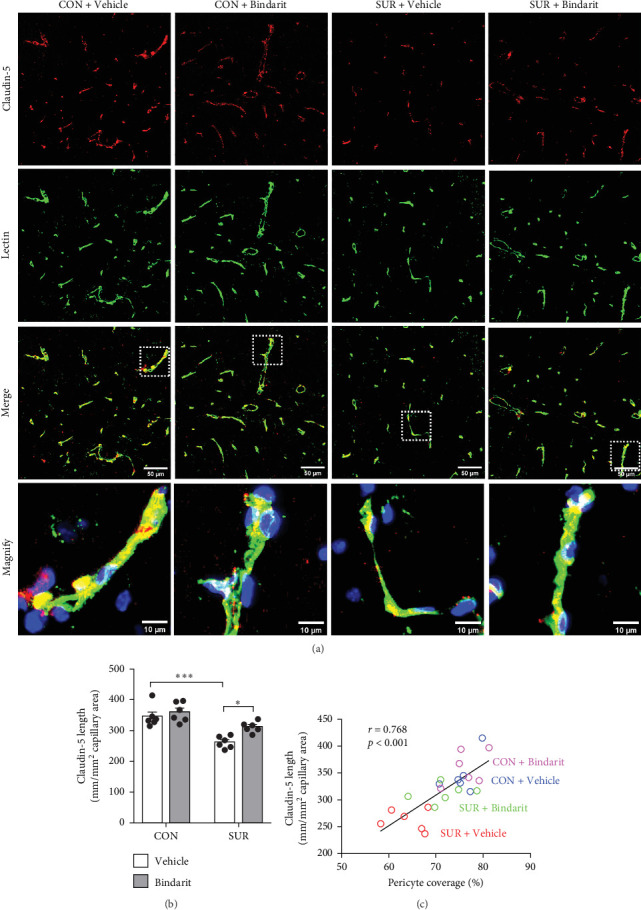
Bindarit reduced the decrease of claudin-5 induced by anesthesia/surgery. (A) Representative confocal images of claudin-5 (red) and lectin-positive capillary (green) in the mice hippocampus. (B) Quantitative analysis of claudin-5 length. (C) Correlation analysis between PC coverage and claudin-5 length. Data are presented as mean ± SEM (*n* = 6), *⁣*^*∗*^*p*  < 0.05, *⁣*^*∗∗∗*^*p*  < 0.001.

**Figure 6:**
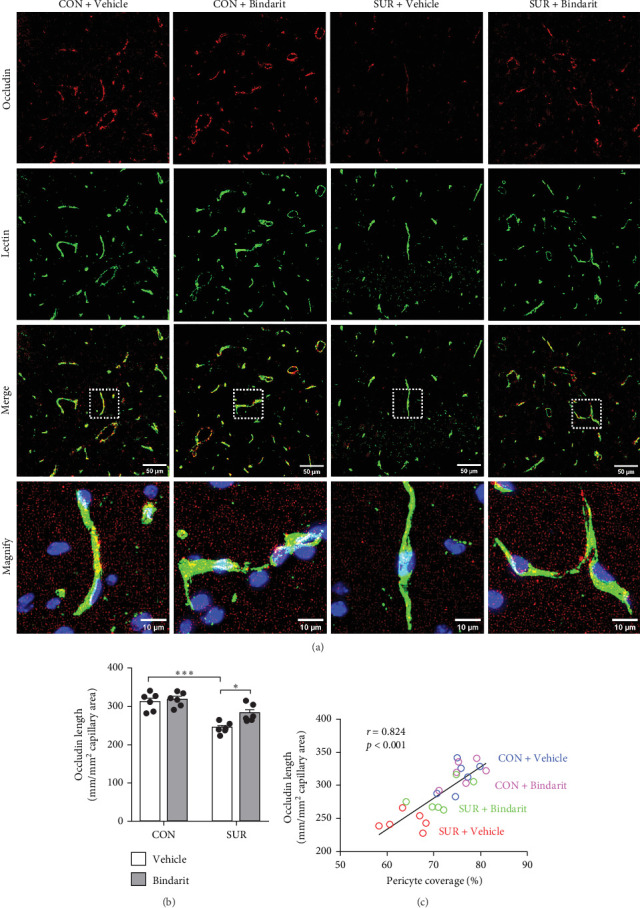
Bindarit reduced the decrease of occludin induced by anesthesia/surgery. (A) Representative confocal images of occludin (red) and lectin-positive capillary (green) in the mice hippocampus. (B) Quantitative analysis of occludin length. (C) Correlation analysis between PC coverage and occludin length. Data are presented as mean ± SEM (*n* = 6), *⁣*^*∗*^*p*  < 0.05, *⁣*^*∗∗∗*^*p*  < 0.001.

TJ proteins are critical components of the BBB. To explore Bindarit's effects on TJ proteins and their relationship with PCs, we measured the lengths of occludin and claudin-5 in the hippocampus using double immunofluorescence (Figures 5A and 6A). The findings demonstrated that the lengths of occludin and claudin-5 in the SUR + Vehicle group were significantly reduced compared to the CON + Vehicle group (Figures 5B and 6B). However, these lengths were notably increased in the SUR + Bindarit group compared to the SUR + Vehicle group (Figures 5B and 6B). Pearson correlation analysis revealed a significant relationship between the decreased length of TJ proteins and PC loss in aged mice after anesthesia/surgery (Figures 5C and 6C).

### 3.5. Bindarit Mitigated BBB Structural Damage Induced by Anesthesia/Surgery in Aged Mice

**Figure 7:**
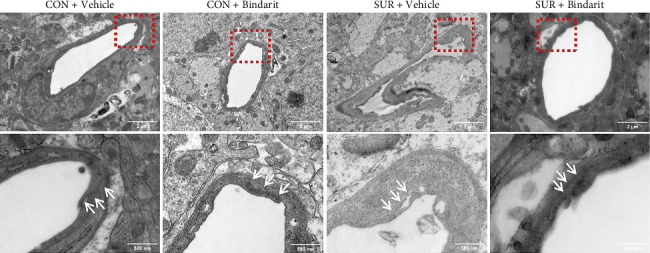
Bindarit alleviated BBB ultrastructure damage induced by anesthesia/surgery. Representative images of hippocampal ultrastructure under TEM in each group of mice, the arrows indicate the TJ.

To further evaluate Bindarit's effect on the BBB, TEM was used to examine the ultrastructure of the BBB (Figure 7). In the control group, the BBB displayed age-related alterations, including mild BBB damageand slight endothelial cell swelling. The basement membrane (BM) was continuous and intact and TJs between endothelial cells were narrow, dense, and tightly connected (Figure 7). The BBB in the SUR + Vehicle group exhibited severe damage: endothelial cells were edematous and flattened; capillaries appeared significantly shrunken and collapsed; local BM structures were fuzzy, discontinuous, and varied in thickness. The gaps in the TJs were widened and the dense areas were shortened (Figure 7). In comparison, BBB damage in the SUR + Bindarit group was less severe, with only slight endothelial cell swelling and slightly blurry local BM structures. The BM was clearer and more continuous with a uniform thickness and the TJs between endothelial cells remained intact, with slightly shorter tight regions and tighter connections (Figure 7).

### 3.6. Bindarit Suppressed the Enhancement of Hippocampal Neuroinflammation in Aged Mice After Anesthesia/Surgery

**Figure 8:**
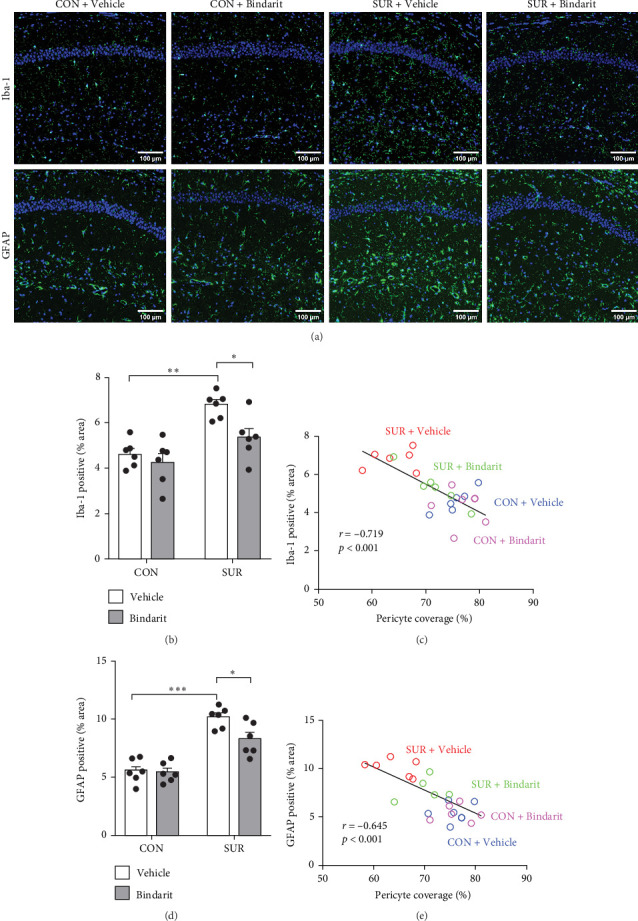
Bindarit reduced the enhanced activation of microglia and astrocytes induced by anesthesia/surgery. (A) Representative confocal images of Iba-1-positive microglia and GFAP-positive astrocytes in the hippocampal CA1 region of mice. (B) Quantitative percentage of the area occupied by Iba-1-positive microglia. (C) Correlation analysis between PC coverage and the activated area of Iba-1-positive microglia. (D) Quantitative percentage of the area occupied by GFAP-positive astrocytes. (E) Correlation analysis between PC coverage and the activated area of GFAP-positive astrocytes. Data are presented as mean ± SEM (*n* = 6), *⁣*^*∗*^*p*  < 0.05, *⁣*^*∗∗*^*p*  < 0.01, *⁣*^*∗∗∗*^*p*  < 0.001.

We employed immunofluorescence to investigate the activation of microglia and astrocytes in the hippocampal CA1 area to evaluate the impact of Bindarit on neuroinflammation and its association with PCs (Figure 8A). The findings demonstrated a significant increase in the activation of microglia and astrocytes in the SUR + Vehicle group compared to the CON + Vehicle group (Figure 8B,D). Conversely, the SUR + Bindarit group exhibited a notable reduction in microglia and astrocyte activation compared to the SUR + Vehicle group (Figure 8B,D). Pearson correlation analysis confirmed a significant relationship between the enhanced activation of microglia and astrocytes following anesthesia/surgery and the loss of PCs (Figure 8C,E).

## 4. Discussion

As aging progresses, the BBB gradually deteriorates [20, 21] and the aging brain becomes increasingly susceptible to chronic inflammation and oxidative stress [22], which diminish its resilience against surgical interventions, thus, heightening the risk of PND [23]. Consistent with our previous research [8, 13], this study utilized aged mice as subjects to mimic the clinical onset of PND through tibial surgery. The results validated the PND model by demonstrating a significant decline in cognitive function in aged mice following anesthesia and surgery. The invasion of peripheral inflammation into the CNS, leading to BBB disruption and neuroinflammation, is identified as a primary mechanism underlying PND [4, 24]. Although prior research has largely focused on the long-term effects of BBB disruption and neuroinflammation [25, 26], less attention has been given to the early mechanisms of PND. This oversight may partially explain the difficulty in identifying effective preventative and therapeutic strategies for PND in clinical settings. Therefore, delineating the sequence of cellular and molecular events in the brain's inflammatory response postanesthesia and surgery, and targeting these early events, is pivotal for guiding the development of interventions for PND. Such efforts are crucial for more effective treatment and improved outcomes in PND management.

CCL2 is a potent chemokine integral to monocyte recruitment during neuroinflammation and is one of the most abundantly expressed chemokines in such conditions [27, 28]. Traditionally, microglia and astrocytes have been recognized as the primary participants in neuroinflammation, with microglia often viewed as the initial responders [29]. However, recent findings by Duan et al. [11] highlight a different paradigm. They demonstrated that PCs release a substantial amount of inflammatory cytokines within 2 h when stimulated by systemic inflammation, faster than endothelial cells, microglia, and astrocytes [11]. Further studies utilizing conditional knockout mice have shown that CCL2 secreted by PCs is the principal contributor to neuroinflammation shortly after brain infection, whereas microglia secreting CCL2 only after 2 h or more [11]. This indicates that PCs are the primary initiators during the early stages of neuroinflammatory processes, with CCL2 serving as the key signaling molecule [30], although other cells also contribute.

Previous research has revealed that PCs detach from the BBB and undergo apoptosis upon inflammatory stimulation [17, 31], a phenomenon also observed in our studies on PND [8]. Additionally, CCL2 expression has been associated with the development of neurological disorders such as multiple sclerosis, Alzheimer's disease, and Parkinson's disease [32]. In this study, we noted a significant increase in hippocampal CCL2 expression following anesthesia and surgery. Treatment with the CCL2 inhibitor Bindarit not only improved cognitive dysfunction and PC coverage but also mitigated brain capillary degeneration associated with PC loss. These findings suggest that Bindarit effectively counters the inflammatory response initiated by early PC-secreted CCL2 in PND, thereby preserving PCs. This underscores the critical role of PC-secreted CCL2 in the pathogenesis of PND.

CNS PCs are crucial components of the BBB, serving as gatekeepers [6, 33]. They are intimately associated with endothelial cells and astrocytic end-feet through the BM, and collaboratively maintaining the BBB's development and the brain's microenvironment balance [34]. Positioned between endothelial cells and astrocytes, PCs play a vital role in preserving BBB integrity [33]. Beyond their structural functions, PCs also engage in direct cell–cell interactions and paracrine signaling with endothelial cells, crucial for maintaining TJs between these cells [35]. Under inflammatory conditions, diminished PC coverage can lead to reduced TJ expression in endothelial cells, compromising BBB integrity [36]. Furthermore, PCs contribute to brain inflammation by producing cytokines, chemokines, and adhesion molecules [31]. Among these, CCL2 is a key chemotactic factor that recruitsmonocytes/macrophages and facilitates the migration of neutrophils and macrophages across the BBB into the CNS. By binding to its main receptor CCR2, CCL2 recruits monocytes and macrophages to the site of injury, and these immune cells release a large number of inflammatory cytokines and matrix metalloproteinases [37], which not only aggravate the local inflammatory response, but also may destroy the structure and function of PC, and exacerbating BBB disruption [37, 38]. After CCL2 binds to CCR2 on PC surface, it activates the downstream NF-κB, MAPK/p38, and JAK/STAT3 pathways, increases the expression of pro-inflammatory cytokines [9, 39], leading to apoptosis or dysfunction of PC. At the same time, CCL2 can affect the interaction between PC and endothelial cells [27], resulting in the shedding of PC from the blood vessel wall, the degeneration of microvessels, the reduction of the integrity of the BBB, and the increase of leakage of plasma proteins, immune cells and toxic substances, resulting in brain edema, inflammation and nerve injury [27]. Additionally, overexpressed CCL2 can damage the TJs of vascular endothelial cells in the normal BBB, potentially via the P38 signaling pathway, leading to further structural damage [40]. In this study, our results demonstrate that anesthesia/surgery significantly resulted in PC loss at the BBB and decreased TJ expression. The administration of the CCL2 inhibitor Bindarit mitigated these effects, indicating that CCL2 secreted by PCs is a critical mediator of BBB disruption in the pathological processes of PND.

Besides BBB disruption, neuroinflammation is also considered a primary pathogenic mechanism of PND [3, 4] and these mechanisms can interact causally, resulting in a mutually reinforcing positive feedback effect [24]. In our findings, anesthesia/surgery significantly enhanced the activation of microglia and astrocytes, correlated with PC loss, and Bindarit attenuated this process. Although perivascular cells and astrocytes are major participants in neuroinflammation [29], they are regulated by PCs. Studies have shown that microglia are often located directly adjacent to PCs in healthy brains [41]. Similarly, microglia aggregation around perivascular cells has been observed in epilepsy models [42], and PCs have been found to promote microglial proliferation and indirectly influence their role in the differentiation of neural stem cells [43]. Additionally, PCs can regulate aquaporins in the end-foot membranes of astrocytes [33], thereby impacting BBB integrity. This regulation may relate to early PC-secreted CCL2 inducing macrophages to cross the BBB and interact with astrocytes and microglia, which in turn causes astrocytes and microglia to secrete more CCL2 and exacerbate neuroinflammation [43]. Moreover, early-stage CCL2 secreted by PCs can directly stimulate microglia and astrocytes, inducing their activation. These observations explain our finding that Bindarit significantly inhibits the excessive activation of microglia and astrocytes associated with reduced PC coverage.

This study had some limitations. First, during neuroinflammation, CCL2 can be secreted by PC, microglia, astrocytes, and endothelial cells in the brain. In this study, we only used western blot to detect the expression of CCL2 on the third postoperative day, thus, we were unable to accurately differentiate the sources of CCL2. In future studies, we will use immunofluorescence costaining (CCL2 with CD13/Iba1/GFAP/CD31) to distinguish the expression of CCL2 in PC, microglia, astrocytes, and endothelial cells. Second, although we have used TEM to observe the ultrastructure of the BBB, we did not directly measure the permeability of the BBB. In future research, we will use tracers to assess the permeability of the BBB. Third, since Bindarit was administered via intraperitoneal injection, its effects may be related to the inflammation induced by the surgical procedure. Therefore, CCL2 in the hippocampal tissue may partially originate from the leakage of blood into the brain. In future studies, we will analyze the expression of cytokines and chemokines in the blood to clarify the expression of CCL2 in the bloodstream postsurgery and the immune modulatory effects of Bindarit beyond the BBB environment. Finally, in this study, we only used CD13 to label PC, which may lead to some inaccuracies in PC measurement. In upcoming studies, we will use more PC-specific markers to ensure the accuracy of PC measurements.

## 5. Conclusions

Overall, our research indicates that suppressing the expression of CCL2 in the hippocampal tissues following anesthesia and surgery can significantly reduce PC loss which might further alleviate BBB disruption and neuroinflammation and protect postoperative cognitive function. Thus, this study implies that the pathological role of PCs through CCL2 in PND, which not only enhancing our understanding of the pathophysiological processes involved in PND but also validating Bindarit might be a potential therapeutic agent for preventing and treating PND.

## Data Availability

The data that support the findings of this study are available from the corresponding author upon reasonable request.
